# Young Risk Takers: Alcohol, Illicit Drugs, and Sexual Practices among a Sample of Music Festival Attendees

**DOI:** 10.1155/2014/357239

**Published:** 2014-12-11

**Authors:** Rebecca Jenkinson, Anna Bowring, Paul Dietze, Margaret Hellard, Megan S. C. Lim

**Affiliations:** ^1^Centre for Population Health, Burnet Institute, 85 Commercial Road, Melbourne, VIC 3004, Australia; ^2^School of Public Health and Preventive Medicine, Monash University, 99 Commercial Road, Melbourne, VIC 3004, Australia

## Abstract

*Background*. Alcohol and other drug use and sexual risk behaviour are increasing among young Australians, with associated preventable health outcomes such as sexually transmissible infections (STIs) on the rise. *Methods*. A cross-sectional study of young people's health behaviours conducted at a music festival in Melbourne, Australia, in 2011. *Results*. 1365 young people aged 16–29 completed the survey; 62% were female with a mean age of 20 years. The majority (94%, *n* = 1287) reported drinking alcohol during the previous 12 months; among those, 32% reported “binge” drinking (6+ drinks) at least weekly. Half (52%) reported ever using illicit drugs and 25% reported past month use. One-quarter (27%) were identified as being at risk of STIs through unprotected sex with new or casual partners during the previous 12 months. Multivariable analyses found that risky sexual behaviour was associated with younger age (≤19 years), younger age of sexual debut (≤15 years), having discussed sexual health/contraception with a doctor, regular binge drinking, and recent illicit drug use. *Conclusion*. Substance use correlated strongly with risky sexual behaviour. Further research should explore young people's knowledge of alcohol/drug-related impairment and associated risk-taking behaviours, and campaigns should encourage appropriate STI testing among music festival attendees.

## 1. Introduction

High-risk alcohol and other drug use are reportedly increasing among young Australians. Findings from population-based surveys suggest that Australians are starting to drink alcohol at an earlier age [1], that the tendency among young adults to drink to intoxication (or “until they cannot remember what happened”) is increasing, and that a substantial minority of young drinkers (around one in five) engage in regular (at least monthly) high-risk drinking (i.e., more than 20 drinks per occasion for young men and more than 11 drinks per occasion for young women) [2, 3]. Likewise, estimates suggest that Australians are among the largest per capita consumers of illicit psychostimulants (ecstasy, methamphetamine, or cocaine) in the world; an estimated 1 in 10 Australians has tried psychostimulants at some point, with well over half a million estimated to use the drugs annually [4, 5].

As recognition of young people's harmful alcohol and other drug use has increased over recent years, so has concern about the sexual health of young Australians. Increasing proportions of young people report being sexually active, and the median age of sexual initiation has declined [6–9]. Young Australians report an increasing number of sexual partners, and (particularly young women) an increasing prevalence of unwanted sex, low rates of condom use, high rates of unplanned pregnancy, and increasing rates of STIs such as chlamydia (the most frequently notified communicable disease in Australia) [8–12]. The most recent National Survey of Australian Secondary Students and Sexual Health found that among sexually active students, one-quarter (23%) reported having had sex with three or more partners during the past year, and half (52%) reported inconsistent condom use (“sometimes” or “never” used condoms) during that time [12].

Information on young people's engagement in risky behaviour typically comes from school-based or household surveys; while these are representative of the general population, the overall prevalence of risk behaviour identified is low (e.g., [5, 13]). The Big Day Out music festival is a useful setting for investigating substance use and sexual risk behaviour within an at-risk population of young people. In this study we examined the sociodemographic characteristics, patterns of alcohol and other drug consumption, and sexual practices of a sample of young music festival attendees in Melbourne, Australia. Additionally, we explored the correlates of engagement in risky sexual practices in this population.

## 2. Materials and Methods

### 2.1. Setting and Recruitment

Participants were recruited at the Melbourne Big Day Out music festival in January 2011 as part of an ongoing behavioural surveillance system, described in detail previously [14, 15]. In brief, approximately 20 trained researchers recruited participants from in and around a market stall at the one-day festival to obtain a convenience sample of young people aged between 16 and 29 years. Eligibility was determined by age, nonintoxication, and sufficient English language skills. Participants were required to self-complete a consent form and short questionnaire taking approximately 10 minutes. Giveaways of bottled water, lollipops, condoms, and health promotion materials, as well as a prize draw to win an Apple iPod, provided incentives for participation. Ethical approval was granted by the Alfred Health Human Ethics Committee, Melbourne, Australia.

### 2.2. Questionnaire

A core set of questions regarding demographics, alcohol consumption, other drug use, and sexual health and behaviour were included in the survey [14]. Alcohol consumption was assessed using a shortened version of the World Health Organisation (WHO) Alcohol Use Disorders Identification Test (AUDIT) [16]: the AUDIT-C [17]. The three AUDIT-C questions provided a measure of alcohol consumption by frequency and volume on a scale of 1 to 12 (among drinkers). “Binge” drinking was defined as six or more drinks in a session on a weekly or more frequent basis during the 12 months prior to survey. The survey also included questions about the use of tobacco (smoking frequency) and the use and injection of illicit drugs.

Sexual health and behaviour questions canvassed sexual identity, condom use with regular, casual, new, and same-sex partners, and health service utilisation. Sexually active participants (ever had vaginal/anal intercourse) were asked about sex with regular partners (boyfriend/girlfriend/partner) and casual partners (all other partner types) during the 12 months prior to interview. A new partner was defined as someone with whom sex had occurred for the first time in the previous three months. An STI test included tests for any infection but excluded pap smears. Condom use was defined as consistent (always used a condom in the past 12 months) or inconsistent (usually, sometimes, or never used a condom in the past 12 months). “Risky sexual behaviour” during the past 12 months was defined as inconsistent condom use with new or casual partners.

### 2.3. Analysis

Questionnaire data were entered into a Microsoft Access database and statistical analysis was conducted in Stata version 11 [18]. Descriptive statistics were used to describe the results of the survey; proportions presented are based on the number of participants responding to a question with missing data excluded. Logistic regression was used to calculate odds ratios (OR), adjusted odds ratios, and corresponding 95% confidence intervals (CI). The candidate correlates for multivariable logistic regression were identified via univariate analysis and existing literature, and the models were refined by a process of backward elimination in which nonsignificant variables were sequentially removed until a subset of wholly significant predictors remained. In all analyses, *P* < 0.05 was considered statistically significant.

## 3. Results

### 3.1. Participants

In total, 1365 surveys were received. The mean age of participants was 20 years (SD 3.36). More females (62%, *n* = 846) than males completed the survey. Participants self-identified as heterosexual (89%), bisexual (6%), gay/lesbian (1%), or questioning (2%). The majority were born in Australia (93%, *n* = 1265), resided in or close to a major city (69%, *n* = 903), and lived with their parents (68%, *n* = 909). Approximately half (44%, *n* = 590 participants) had completed or were in the process of completing post-high school education.

### 3.2. Alcohol Consumption and Related Risk

Almost all participants (94%, *n* = 1287) reported drinking alcohol during the 12 months prior to survey, with premixed spirits (58%), bottled spirits/liqueurs (55%), regular strength beer (44%), and bottled wine (27%) the beverage types most commonly consumed. The median AUDIT-C score among drinkers was 6 (IQR 1, 12), with 32% (*n* = 403) reporting “binge” drinking (6+ drinks) on a weekly or more frequent basis during the previous 12 months, and 21% (*n* = 269) reporting drinking 20+ drinks on at least one occasion during that time.

Experience of alcohol-related harm was also reported: 54% (*n* = 692) of all drinkers reported at least one occasion during the previous 12 months when they had been unable to remember what happened the night before because of their drinking, and around 18% (*n* = 236) of participants reported that they or someone else had been injured as a result of their drinking during that time.

### 3.3. Other Drug Consumption

Almost one-third (32%, *n* = 435) reported currently smoking cigarettes or other tobacco; 68% of those on a weekly or more frequent basis. Half (52%, *n* = 700) also reported lifetime use of illicit drugs, with 25% (*n* = 335) of all participants reporting illicit drug use during the month prior to interview. The most commonly used illicit drugs among recent users included cannabis (84%); speed (35%); LSD/mushrooms (35%); ecstasy (30%); cocaine (17%); and benzodiazepines (14%).

### 3.4. Sexual Practices

Most participants (84%, *n* = 1142) reported being sexually active; the mean age of sexual debut was 16 years (SD 2.16), with 32% (*n* = 350) reporting an age of sexual debut of 15 years or younger. Among sexually active participants, 87% (*n* = 943) reported having had sex with a regular partner, 55% (*n* = 601) reported sex with a casual partner, 44% (*n* = 497) reported sex with two or more partners, and 9% (*n* = 93) reported sex with a same-sex partner in the previous 12 months. One-third (35%, *n* = 387) also reported having had sex with a “new” partner during the three months prior to interview.

One-quarter (27%, *n* = 309) of all sexually active participants reported having had unprotected sex with new or casual partners during the 12 months prior to survey (defined in this study as risky sexual behaviour). While close to half (43%, *n* = 490) reported having spoken to a general practitioner (GP) about sexual health and/or contraception during that time, only one-third (34%, *n* = 388) reported having ever been tested for sexually transmissible infections (STIs) and one-quarter (24%, *n* = 279) reported having been tested during the previous 12 months.

### 3.5. Correlates of Risky Sexual Behaviour

In univariate analyses, correlates of risky sexual behaviour during the past 12 months included (Table 1) younger age (16–19 years compared to 20–29 years); not yet having undertaken or completed post-high school education; and greater weekly spending money for recreational activities (≥AUD$80 compared to <AUD$80). In regard to sexual health and behaviour, correlates included younger age of sexual debut (≤15 years compared to >15 years); same-sex partner during the 12 months prior to survey; having discussed sexual health/contraception with a GP during the 12 months prior to survey; and having ever had an STI test. Risky sexual behaviour was also correlated with alcohol and other drug use practices in univariate analyses: participants who currently smoked (OR = 1.82, 95% CI 1.39–2.38, *P* < 0.001); engaged in regular binge drinking (OR = 2.23, 95% CI 1.70–2.93, *P* < 0.001); or engaged in recent illicit drug use (OR = 1.82, 95% CI 1.37–2.41, *P* < 0.001) were all more likely to report engagement in risky sexual behaviour during the 12 months prior to survey (Table 1).

In the final multivariable analysis, STI risk behaviour remained significantly and independently associated with younger age (OR = 1.54, 95% CI 1.14–2.07, *P* < 0.01), younger age of sexual debut (OR = 1.70, 95% CI 1.26–2.31, *P* < 0.01), regular (at least weekly) binge drinking (OR = 2.13, 95% CI 1.58–2.88, *P* < 0.001), and recent (past month) illicit drug use (OR = 1.68, 95% CI 1.22–2.30, *P* < 0.01). Having discussed sexual health and/or contraception with a GP in the previous 12 months was also associated with engagement in risky sexual behaviour (OR = 1.58, 95% CI 1.18–2.11, *P* < 0.01).

## 4. Discussion

Engagement in risky substance use and sexual behaviour was common among this sample of young Australian music festival attendees. Drinking alcohol was clearly central to their lives; 94% reported having consumed alcohol during the 12 months prior to interview, most commonly spirits (premixed and bottled) and regular strength beer. Half of the sample reported having consumed 11 or more drinks on at least one occasion during the previous 12 months and one-third reported regular (at least weekly) “binge” drinking during that time. The young people in this study reported drinking at levels well above those recommended in national alcohol guidelines [19, 20], and according to their (past 12 months) AUDIT-C consumption score, at levels that would place them at risk for alcohol-related harm. Furthermore, consistent with previous research suggesting that drug use is becoming increasingly normalised in populations of young Australians involved in music subcultures [21–24], the prevalence of illicit drug use among this population of young people was much higher than in the general community; half of all participants reported lifetime illicit drug consumption and one in four reported consumption during the month prior to interview, highlighting an increased risk for negative drug-related physical and psychological consequences in this group.

The majority of young people who participated in this study were sexually active; while one in three of these participants reported having ever been screened for STIs (fewer during the 12 months prior to survey), a substantial proportion were identified as being at risk of an STI through recent (past 12 months) unprotected sex with new or casual partners. Given the increasing prevalence of STIs among sexually active young people in Australia, and in turn, the potential for longer-term health problems, national guidelines recommend annual STI screening for those aged 15–29 years [25]; however, findings from this study suggest that a large proportion of this subpopulation are not being regularly screened and remain at risk of STI acquisition and associated consequences.

The factors that were significantly and independently correlated with engagement in risky sexual behaviour included younger age, younger age of sexual debut, having discussed sexual health/contraception with a GP, regular binge drinking, and recent illicit drug use. These findings support other research that has found associations between younger age of sexual debut and risky sexual behaviour (e.g., [26–28]). Further, while investigations into the association between substance use and sexual risk-taking have produced mixed findings, with a myriad of factors complicating the relationship, including drug consumption patterns (i.e., frequency and quantity of use) and motivations for use, “binge” alcohol consumption has commonly been associated with engagement in risky sexual behaviour (such as multiple sex partners and infrequent condom use) (e.g., [29–31]). Cross-sectional research has also found psychostimulant use to be associated with riskier sexual practices across a range of populations, highlighting the correlation between the two types of behaviour (e.g., [32–37]). Though temporal relationships between these types of behaviour cannot be ascertained from this study and causal relationships cannot be determined, our findings are indicative of an association between heavier patterns of alcohol and other drug consumption (which may result in some level of alcohol or drug-related impairment and impaired decision-making) and engagement in sexual risk behaviour among young people.

This study is limited by convenience sampling and findings are therefore not generalisable to all young Australians. Prior surveys have demonstrated that participants recruited at the Melbourne Big Day Out music festival are more likely to engage in alcohol-, drug-, and sex-related risk behaviour than other young Australians [38, 39] and may also be of a higher socioeconomic status given admission costs (AUD$164 in 2011). Second, although there is evidence that self-reported data on sensitive behaviour (such as illicit drug use) tends to be reliable if confidentiality is assured (e.g., [40–43]) the participants' responses may have been affected by social desirability bias. Third, the findings reported here may be subject to some recall bias (most likely underestimation or underreporting), given the timeframe enquired about.

Nevertheless, music festivals such as the Big Day Out provide an excellent opportunity to investigate young people's engagement in a range of risk behaviours and to improve knowledge about behaviours that may place young people at risk of harm. The Melbourne Big Day Out surveys have previously served as a platform for a range of other research and intervention studies, including a randomised controlled trial of the impact of sexual health promotion via email and mobile phone messages that found an improvement in STI knowledge among participants [44], and as an opportunity to evaluate national sexual health and binge drinking awareness campaigns [45, 46] and alcohol guidelines [47].

## 5. Conclusion

Heavy patterns of alcohol and other drug use were some of the strongest correlates of young people's engagement in risky sexual behaviour. The complex relationship between drug use and sexual risk-taking requires further investigation, given that unprotected sex raises concerns about the transmission of STIs and unplanned pregnancy. Further research should explore young people's knowledge and understanding of alcohol and drug-related impairment and associated risk-taking behaviour, and education and harm reduction campaigns should consider targeting young music festival attendees with specific health promotion messages in regard to safer alcohol and other drug use and sex practices and to encourage appropriate STI testing.

## Figures and Tables

**Table 1:** Correlates of risky sexual behaviour during the past 12 months (*n* = 1142).

Engaged in risky sexual behaviour^+∧^	No (*n* = 833)	Yes (*n* = 309)	Unadjusted OR (95% CI), *P*	Adjusted OR (95% CI), *P*
	*n* (%)	*n* (%)		
Gender				
Female	509 (74)	179 (26)	1.0	
Male	324 (71)	130 (29)	1.14 (0.87, 1.49), 0.33	
Age (years)				
16–19	404 (70)	176 (30)	1.40 (1.08, 1.83), 0.01	1.54 (1.14, 2.07), <0.01
20–29	429 (76)	133 (24)	1.0	1.0
Postcode				
Metropolitan	533 (73)	196 (27)	1.0	
Rural/regional	260 (72)	100 (28)	1.05 (0.79, 1.39), 0.76	
Country of Birth				
Australia	769 (73)	288 (27)	1.12 (0.67, 1.87), 0.66	
Elsewhere	63 (75)	21 (25)	1.0	
Completed or undertaking post-high school education				
No	411 (71)	171 (29)	1.30 (1.00, 1.69), 0.05	
Yes	415 (76)	133 (24)	1.0	
Live with parents				
No	309 (75)	511 (72)	1.0	
Yes	511 (72)	201 (28)	1.17 (0.89, 1.54), 0.27	
Recreational income (per week)				
<A$80	354 (77)	108 (23)	1.0	
≥A$80	459 (70)	193 (30)	1.38 (1.05, 1.81), 0.02	
Consumed alcohol^#^				
No	20 (83)	4 (17)	1.0	
Yes	803 (73)	300 (27)	1.17 (0.89, 1.54), 0.27	
Binge (≥6 drinks in a session)^#∗^				
<weekly	560 (79)	151 (21)	1.0	1.0
≥weekly	238 (62)	143 (38)	2.23 (1.70, 2.93), <0.001	2.13 (1.58, 2.88), <0.001
Currently smoke tobacco				
No	575 (77)	169 (23)	1.0	
Yes	256 (65)	137 (35)	1.82 (1.39, 2.38), <0.001	
Used illicit drugs in past month				
No	629 (77)	191 (23)	1.0	1.0
Yes	200 (64)	111 (36)	1.82 (1.37, 2.41), <0.001	1.68 (1.22, 2.30), <0.01
Age first sex (years)				
≤15	219 (63)	131 (37)	2.09 (1.58, 2.76), <0.001	1.70 (1.26, 2.31), <0.01
>15	577 (78)	165 (22)	1.0	1.0
Reported any same-sex partners^#^				
No	747 (75)	247 (25)	1.0	
Yes	50 (54)	43 (46)	2.60 (1.69, 4.01), <0.001	
Discussed sexual health and/or contraception with GP^#^				
No	482 (75)	157 (25)	1.0	1.58 (1.18, 2.11), <0.01
Yes	343 (70)	147 (30)	1.32 (1.01, 1.71), 0.04	1.0
Ever had STI test				
No	559 (75)	186 (25)	1.0	
Yes	270 (70)	118 (30)	1.31 (1.00, 1.72), 0.05	

^+^Risky sexual behaviour was defined as inconsistent condom use with new or casual partners during the past 12 months.

^∧^Among those who reported having ever had sex.

^#^During past 12 months.

^*^Among those who reported drinking alcohol during past 12 months.
